# Patient activation and patient-reported outcomes of men from a community pharmacy lifestyle intervention after prostate cancer treatment

**DOI:** 10.1007/s00520-021-06404-5

**Published:** 2021-07-21

**Authors:** Agnieszka Lemanska, Karen Poole, Ralph Manders, John Marshall, Zachariah Nazar, Kevin Noble, John M. Saxton, Lauren Turner, Gary Warner, Bruce A. Griffin, Sara Faithfull

**Affiliations:** 1 School of Health Sciences, Faculty of Health and Medical Sciences, University of Surrey, Guildford, UK; 2 Department of Nutritional Sciences, Faculty of Health and Medical Sciences, University of Surrey, Guildford, UK; 3 Patient and Public Involvement, Prostate Cancer UK, London, UK; 4 Clinical Pharmacy and Practice Department, College of Pharmacy, QU Health, Qatar University, Doha, Qatar; 5 Pinnacle Health Partnership LLP, East Cowes, Isle of Wight UK; 6 Department of Sport, Health and Exercise Science, Faculty of Health Sciences, University of Hull, Hull, UK; 7 Frimley Health NHS Foundation Trust, Frimley, Surrey UK

**Keywords:** Patient-reported outcomes (PROs), Prostate cancer, Lifestyle interventions, Community pharmacy

## Abstract

**Purpose:**

To report patient activation, which is the knowledge, skills, and confidence in self-managing health conditions, and patient-reported outcomes of men after prostate cancer treatment from a community pharmacy lifestyle intervention.

**Methods:**

The 3-month lifestyle intervention was delivered to 116 men in nine community pharmacies in the UK. Patient Activation Measure (PAM) was assessed at baseline, 3 and 6 months. Prostate cancer-related function and quality of life were assessed using the European Prostate Cancer Index Composite (EPIC-26) and EuroQOL 5-dimension 5-level (EQ5D-5L) questionnaires at baseline and 6 months. Lifestyle assessments included Mediterranean Diet Adherence Screener (MEDAS) at baseline, 3 and 6 months and Godin Leisure Time Exercise Questionnaire (GLTEQ) at baseline and 3 months.

**Results:**

PAM score increased from 62 [95% CI 59–65] at baseline to 66 [64–69] after the intervention (*p* = 0.001) and remained higher at 6 months (*p* = 0.008). Scores for all the EPIC-26 domains (urinary, bowel and hormonal) were high at both assessments, indicating good function (between 74 [70–78] and 89 [86–91]), except sexual domain, where scores were much lower (21 [17–25] at baseline, increasing to 24 [20–28] at 6 months (*p* = 0.012)). In EQ5D-5L, 3% of men [1–9] reported self-care problems, while 50% [41–60] reported pain and discomfort, and no significant changes over time. Men who received androgen deprivation therapy, compared with those who did not, reported higher (better) urinary incontinence scores (*p* < 0.001), but lower (worse) scores in the urinary irritative/obstructive (*p* = 0.003), bowel (*p* < 0.001) and hormonal (*p* < 0.001) domains. Poor sexual function was common across all age groups irrespective of prostate cancer treatment.

**Conclusions:**

The intervention led to significant improvements in patient activation, exercise and diet. Community pharmacy could deliver effective services to address sexual dysfunction, pain and discomfort which are common after prostate cancer.

**Supplementary Information:**

The online version contains supplementary material available at 10.1007/s00520-021-06404-5.

## Introduction

Prostate cancer treatments, and specifically androgen deprivation therapy (ADT), have been shown to negatively affect prostate cancer-related function and quality of life [1]. Lifestyle interventions are needed to reduce adverse effects of prostate cancer and its treatment [2]. In addition, because most men (80%) live 10 years or more after cancer diagnosis, and prostate cancer is now classed as a long-term condition, men are more likely to require greater support from primary care and community health and wellbeing services for their long-term needs [3].

In the UK, men are normally discharged from cancer centres to the primary care follow-up system 2 years after completion of their primary treatment [3]. Therefore, community health and wellbeing services, and particularly community pharmacies, could become important points of contact for men to consult about erectile dysfunction, urinary, bowel or hormonal problems such as weight gain, depression or lack of energy which are common after prostate cancer [4]. As outlined in the long-term plan for the primary care reform in the UK (2019) [5], further research and policy changes are needed to optimise the role of community pharmacies in addressing long-term health-related needs of cancer survivors. More patient-reported outcomes research is also needed. This is to demonstrate the impact of prostate cancer on quality of life and functional outcomes of menwho are living in the community.

Traditionally, community pharmacies have been perceived primarily as retailers and services to dispense medications. However, the increasing complexity of healthcare needs of an aging population and increasing demands on healthcare services, with a shift towards patient-centred care models, have expanded the role of the community pharmacy worldwide [6, 7]. The community pharmacy sector is now more involved in health promotion, such as smoking cessation and weight loss [8], provision of advanced services such as vaccinations [9], management of long-term conditions such as asthma [10, 11] and cancer awareness and screening [12, 13]. Accessibility (pharmacies are often located on high streets), no need for an appointment, and highly trained clinical staff make the community pharmacy well placed to become more involved in cancer care [7].

In 2019, we published results from a feasibility study of a 3-month community pharmacy-based lifestyle intervention aimed at improving the health behaviours of men after prostate cancer treatment [14]. The intervention tested a novel delivery approach via community pharmacies and to our knowledge is the first community pharmacy intervention developed and implemented to support men after prostate cancer treatment. Here, we report on patient activation, lifestyle behaviours and patient-reported outcomes (PROs) for health domains related to prostate cancer, measured at three time points, before and after the intervention, and at 6 months follow-up. We also undertook a subgroup analysis of baseline PROs according to age and ADT treatment.

## Methods

### Study design, participants, and the community pharmacy intervention

The community pharmacy lifestyle intervention called TrueNTH Exercise and Diet (https://prostatecanceruk.org/about-us/projects-and-policies/truenth), funded by the Movember Foundation and Prostate Cancer UK, was developed and tested in nine community pharmacies in the Portsmouth area of the UK. The design is reported elsewhere [14], but in summary, pharmacy teams were trained to deliver a health assessment including fitness, strength and anthropometric measures. Based on this health assessment, a bespoke computer algorithm generated a personalised lifestyle prescription for patients to implement at home. Participants received the prescription, verbal advice from a pharmacist and a pack of resources that included an educational DVD, a booklet with physical activity and healthy eating advice (including recipes), resistance bands for strength exercises and a pedometer to measure step count. Support was provided by two telephone calls from a pharmacist at weeks 1 and 6. Men were reassessed 3 months later at a second pharmacy consultation. Details of the intervention, its components, timelines and primary results can be found elsewhere [14]. PROs were administered via postal questionnaires at baseline, 3 and 6 months.

This was a non-randomised, single-group study. Pharmacy teams delivered the intervention to all men recruited from one cancer centre between June 2016 and April 2017. Men with non-metastatic prostate cancer who had completed their primary treatment at least 3 months before (6 months for brachytherapy) were recruited if they had at least one of three cardiovascular risk factors: overweight or obese (body mass index [BMI] ≥ 25), on active ADT and/or diagnosed hypertension. Physically active men, achieving the UK’s Chief Medical Officer guidelines [15] of a minimum 150 min of moderate physical activity or a minimum of 75 min of vigorous physical activity per week, and those with an underlying medical condition that would limit their capacity to respond to diet and exercise advice, were excluded. Detailed methods and participant recruitment information are reported elsewhere [14].

### Data collection procedures

**Fig. 1:**
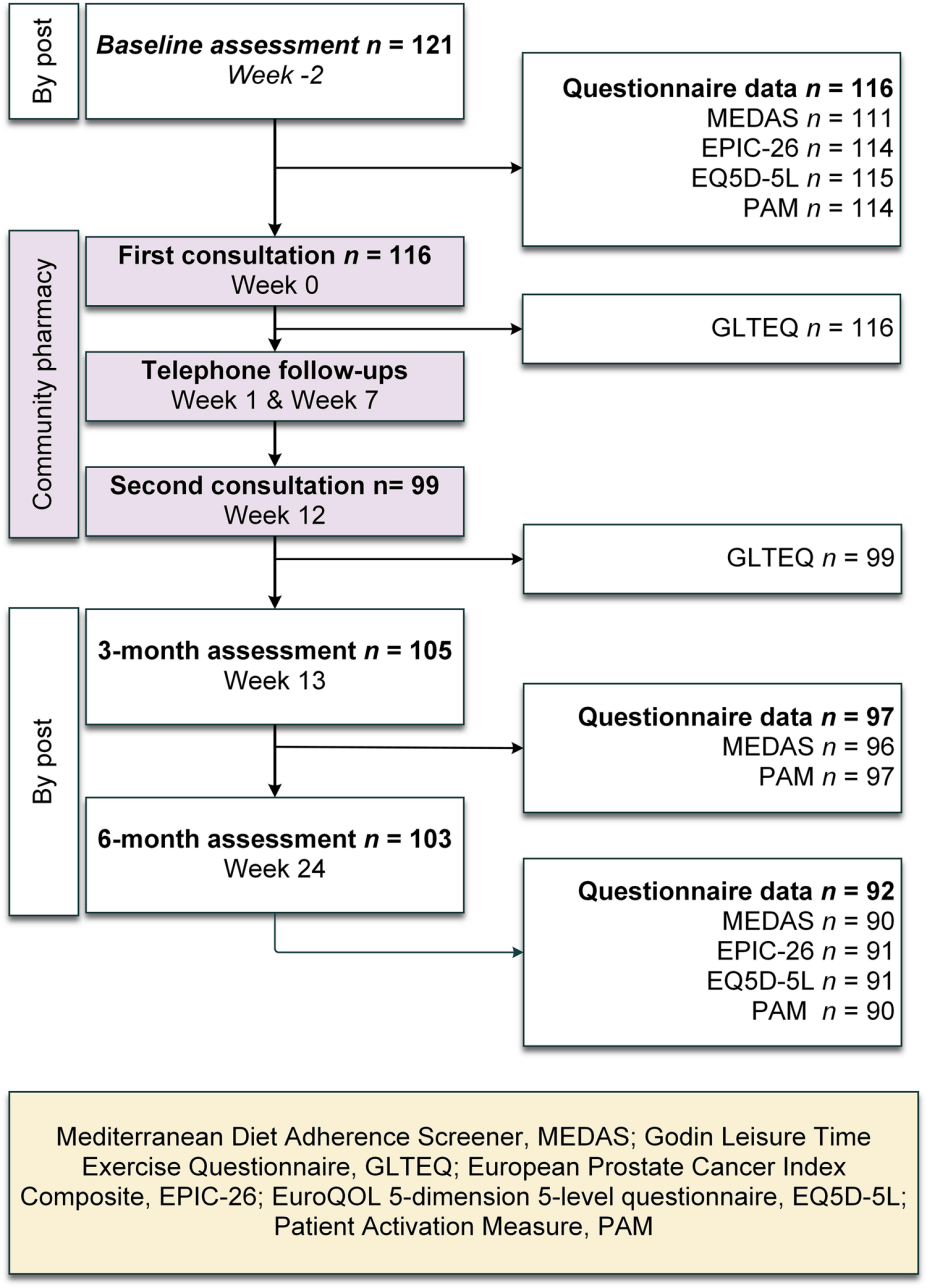
Consort diagram indicating the number of participants completing assessments and patient-reported outcome measures at each point in time

The baseline assessment took place before the start of the intervention. Men were sent a questionnaire pack (Appendix 1 in the Supplementary information) by post, which included a standard set of validated measures developed for the assessment of function and quality of life in this population [16]. The pack also included validated measures to assess patient activation, diet and physical activity. After completion of the community pharmacy intervention, postal questionnaires were completed at 3 months from baseline to assess changes (Appendix 2 in the Supplementary information) and at 6 months from baseline (Appendix 3 in the Supplementary information) to assess the longer-term sustainability of changes. The CONSORT diagram (Fig. 1) shows the timelines for data collection and the number of participants that completed each component.

### Patient activation

A 13-item version of the Patient Activation Measure (PAM) was used to assess knowledge, role and confidence in managing one’s own health [17]. Participants rated their answers on a scale of 1–5 according to their agreement with a health management statement. Using a PAM scoring table, the answers were transformed into a composite PAM score for each participant ranging from 0 to 100, with 100 representing the highest level of activation, and into a four-level patient activation variable, which ranged from level 1 that represented ‘passive’ and level 4 that represented ‘proactive’ [18].

### Healthy lifestyle behaviours

Diet was assessed with the 14-item Mediterranean Diet Adherence Screener (MEDAS) questionnaire [19, 20]. MEDAS consists of 12 items that target information about food consumption and 2 items about food intake habits. Each question is scored 0 or 1, so the total MEDAS score ranges from 0 to 14, with a higher score indicating better adherence to the Mediterranean diet. Unanswered items were considered to indicate non-consumption and scored as 0. A 3-level variable was used to categorise people into not adherent (MEDAS score < 7), mid-range value for adherence (7–8) and strict adherence (≥ 9) [21].

Self-reported physical activity was assessed by the Godin leisure-time exercise questionnaire (GLTEQ) [22, 23]. Total score was calculated by multiplying the weekly frequency of strenuous, moderate and mild physical activities by 9, 5 and 3 respectively, and summing the weighted components.

### Patient-reported outcome measures

A total of 26 prostate cancer-related functional outcomes were measured with the Expanded Prostate Cancer Index Composite Short Form (EPIC-26) tool [24], and arranged in five multi-item health domains: urinary incontinence, urinary irritation and obstruction and bowel, hormonal and sexual function. Each outcome was scored by participants on a 4- or 5-point Likert scale. Summary scores for each domain were calculated on a scale from 0 to 100, with 100 representing the best possible function, as recommended by scoring guidelines [25].

Quality of life was evaluated with the Euro quality of life 5-dimension 5-level (EQ5D-5L) questionnaire [26]. This records information on quality of life with five single-item domains: mobility, self-care, usual activities, pain and discomfort and anxiety and depression. These were scored by participants on a 5-point Likert scale, with 1 representing no problem and 2–5 representing severity of the problem (slight, moderate, severe and extreme problem, respectively). As recommended by the user guide [27], index values summarising scores across domains were derived from the English value set [28]. General health status was also scored by participants on a sliding scale 0 to 100, with 100 representing the best imaginable state of health.

### Statistical analysis

For men who did not complete a questionnaire or did not answer enough questions in a questionnaire for a valid score, missing data were not imputed, and summary scores were calculated in accordance with instructions that accompany each tool. Results for participants with available and valid data were included in a complete case analysis approach. Figure 1 shows the number of participants with valid data at each time point.

The study sample was summarised using means and 95% CI for normal data, medians and 95% CI for skewed data and percentages and 95% CI for categorical data. Summary statistics were chosen to enable comparisons with other published studies. To test changes over time in outcomes and for differences between age and ADT groups at baseline, statistical tests appropriate to the type of data were used, including t-test, ANOVA, Wilcoxon signed rank, Chi-squared and McNemar tests (the specific information on which test was used is detailed in tables and figures). Data were imputed into Excel and quality checked. All the pre-processing and statistical analyses were performed in R statistical software version 4.0.2 (R Foundation for Statistical Computing, Vienna, Austria). Statistical significance was set at *p* < 0.05. Because statistical significance may be different from clinical relevance, where available, the results were interpreted in relation to previously published, minimally important clinical differences.

## Results

Of 121 men who participated in the baseline assessment, 118 men returned their postal questionnaire, and 116 attended the first community pharmacy consultation. The analyses were based on 116 participants (mean age 70.4 ± 7.2) who commenced the intervention and were on an intention to treat basis. Participant baseline characteristics are summarised in Table 1 and the number of participants providing data at each timepoint is shown in Fig. 1. Only 99 participants attended the second pharmacy consultation at 3 months. However, some men who did not attend the second pharmacy visit, continued to provide postal questionnaires at 3 and 6 months. Of the total 116, 11 (9%) participants were lost to follow-up at 3 months, and a further 2 at 6 months, bringing the total drop-out rate to 13 (11%).

**Table 1:** Summary baseline characteristics of the study population (*N* = 116)

	Mean (SD)	Median (IQR: Q1, Q3)	*n* (%)
Age (years)	70.4 (7.2)	71 (65, 76)	
< 65			25 (22)
65–74			57 (49)
≥ 75			34 (29)
Pharmacy (site)			
A			21 (18)
B1			9 (8)
B2			8 (7)
B3			9 (8)
B4			18 (16)
C1			30 (26)
C2			8 (7)
C3			4 (3)
C4			9 (8)
Ethnicity			
White			114 (98)
Black			2 (2)
Marital status			
Married/partner			102 (88)
Single/widowed			14 (12)
Retirement			
Retired			89 (77)
Working			27 (23)
Smoking status (current smoker)			
Non-smoker			61 (53)
Ex-smoker			53 (46)
Current smoker			2 (2)
Index of multiple deprivation (IMD)		7.5 (5, 9)	
1–3 (most deprived)			16 (14)
4–6			24 (21)
7–8			37 (32)
9–10 (least deprived)			39 (34)
Time since diagnosis (years)	1.5 (0.7)	1.5 (0.9, 2.1)	
≤ 1 year			72 (62)
> 1 year			36 (31)
Missing			8 (7)
Treatment			
Surgery			49 (42)
Radiotherapy			69 (60)
Brachytherapy			4 (4)
Androgen deprivation therapy (ADT)			66 (57)

At 3 months, the PAM score increased on average by 4 points from 62 (95% CI 59 to 65) to 66 (95% CI 64 to 69). This improvement in patient activation was statistically significant (*p* = 0.001), clinically relevant [29] and sustained at 6 months (*p* = 0.008). There was also a statistically significant increase in physical activity. GLTEQ score increased from 36 (95% CI 29 to 43) at baseline to 42 (95% CI 37 to 48) after the intervention (*p* = 0.010). The median MEDAS score was 6 (95% CI 6 to 7) at baseline and increased to 7 (95% CI 7 to 8) at 3 months (*p* = 0.003), an improvement that was sustained at 6 months (*p* < 0.001). This improvement in diet after the intervention could be considered clinically significant because participants moved to higher levels of adherence (Table 2). For example, the percentage of men in the strict adherence category increased from 14% (95% CI 8 to 22) at baseline to 26% (95% CI 17 to 36, *p* = 0.003) at 3 months and to 28% (95% CI 18 to 40, *p* = 0.002) at 6 months.

**Table 2:** The analysis of change over time in patient-reported outcomes and lifestyle measures

	Baseline	3 Months	p Value	6 Months	*p* Value
Lifestyle assessments					
PAM					
Score^c^	62 (59–65)	66 (64–69)	0.001^c^	66 (63–69)	0.008^c^
Level 1 (passive)^b^	13 (8–21)	5 (2–12)	0.081^a^	8 (3–15)	0.089^a^
Level 2^b^	14 (8–22)	12 (7–21)		13 (7–22)	
Level 3^b^	51 (41–60)	55 (44–65)		47 (37–58)	
Level 4 (active)^b^	22 (15–31)	28 (19–38)		32 (22–42)	
MEDAS					
Score^a^	6 (6–7)	7 (7–8)	0.003^a^	7 (7–8)	< 0.001^a^
MEDAS score < 7 (no adherence)^b^	52 (43–62)	33 (24–43)	0.003^a^	32 (22–45)	0.002^a^
MEDAS score 7–8 (moderate adherence)^b^	33 (25–43)	42 (32–52)		39 (28–52)	
MEDAS score ≥ 9 (strict adherence)^b^	14 (8–22)	26 (17–36)		28 (18–40)	
GLTEQ^c^	36 (29–43)	42 (37–48)	0.010^c^		
PROMs					
EPIC-26 domains					
Urinary incontinence^c^	83 (79–87)			82 (78–86)	0.364^c^
Urinary irritative and obstructive^c^	88 (86–91)			89 (86–91)	0.692^c^
Bowel function^c^	88 (85–92)			88 (85–92)	0.923^c^
Hormonal function^c^	74 (70–78)			77 (73–80)	0.006^c^
Sexual function^c^	21 (17–25)			24 (20–28)	0.012^c^
EQ5D-5L items					
Mobility^b^	32 (23–43)			25 (17–36)	0.114^b^
Self-care^b^	3 (1–9)			3 (1–9)	1.000^b^
Usual activities^b^	35 (25–46)			25 (17–36)	0.052^b^
Pain and discomfort^b^	50 (39–60)			48 (38–59)	1.000^b^
Anxiety and depression^b^	30 (21–40)			28 (19–38)	0.803^b^
Summary index value^c^	0.891 (0.865–0.917)			0.903 (0.878–0.928)	0.160^c^
Summary index value = 1 (full health state)^b^	35 (25–46)			40 (30–50)	0.522^b^
Health status (sliding scale 0 to 100)^c^	79 (76–81)			81 (78–83)	0.182^c^

*PAM* patient activation measure as a continuous score and categorical 4-level variable; *MEDAS* Mediterranean diet adherence screener; *GLTEQ* Godin leisure time exercise questionnaire; *EPIC-26* European prostate cancer index composite 26-tem short form: urinary incontinence, urinary irritative and obstructive, bowel function, hormonal function and sexual function domains standardised to a scale of 0–100; *EQ5D-5L* Euro quality of life 5-dimension 5-level: mobility, self-care, usual activities, pain and discomfort, anxiety and depression domains expressed as dichotomous variables (no symptom vs any symptom severity), and summary index value and health status as a continuous score. All variables rescaled so that a higher score represents a better outcome. Data are presented as median scores and 95% confidence intervals (CI) denoted by ^a^, percentages (%) of men and 95% CI denoted by ^b^ and mean scores and 95% CI denoted by ^c^. Statistical significance *p* was estimated accordingly to the data type with the Wilcoxon signed rank test (*p* value denoted by ^a^), the McNemar test (*p*-value denoted by ^b^) and the paired t-test (*p*-value denoted by ^c^)

For the whole sample, mean EPIC-26 scores for domains such as urinary incontinence, urinary irritation and obstruction, bowel function and hormonal function were high at both time points (ranged between 74 [95% CI 70 to 78] and 89 [95% CI 86 to 91], Table 2). This indicated good function in all EPIC-26 domains except in sexual function, where the mean scores were relatively low, 21 (95% CI 17 to 25) at baseline and 24 (95% CI 20 to 28) at 6 months. The 3-point improvement in sexual function at 6 months was statistically significant (*p* = 0.012), but not relevant clinically (10–12 points improvement in the sexual domain would be considered clinically relevant [30]). We also observed a 3-point improvement in hormonal function. This was statistically significant (*p* = 0.006) and could potentially reach levels of clinical relevance for some patients because the 95% CI for the average difference was 1 to 5, and the previously estimated minimally important difference for the EPIC-26 hormonal domain was 4–6 points [30].

**Fig. 2:**
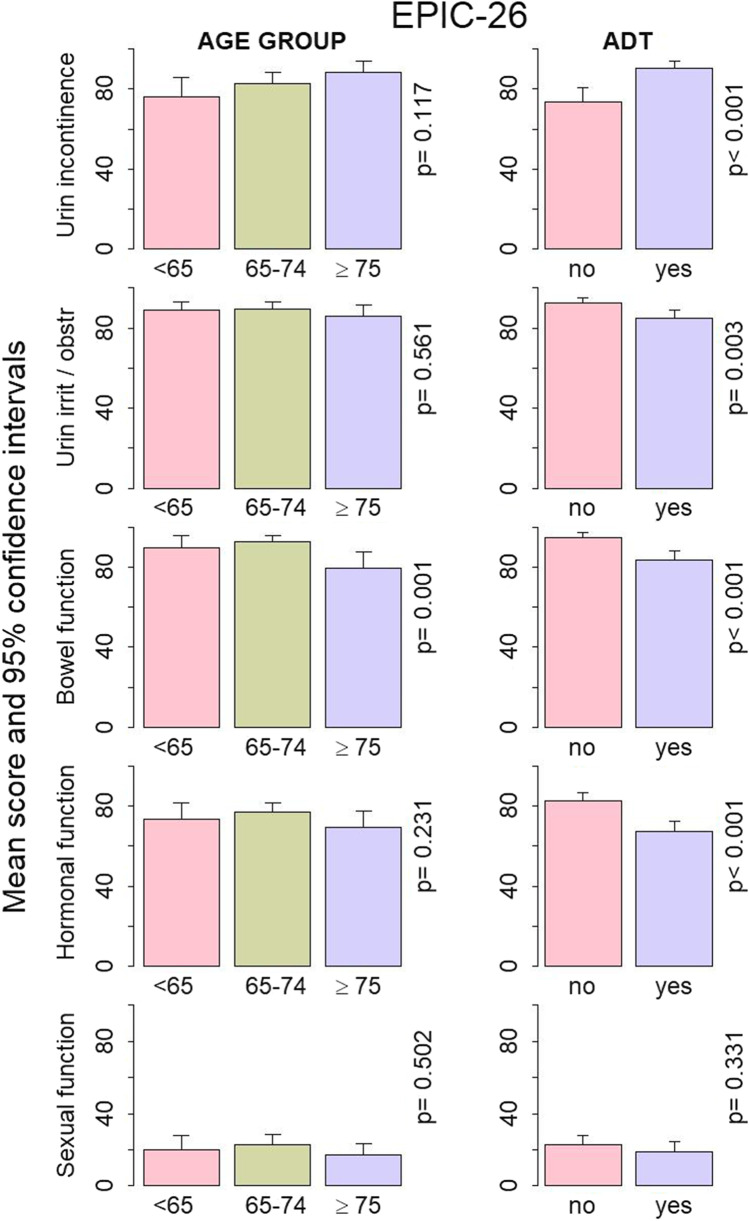
Baseline mean scores and 95% confidence intervals for the five EPIC-26 domains: urinary incontinence, urinary irritative/obstructive, bowel function, hormonal function and sexual function by age group and by androgen deprivation therapy (ADT) (yes/no). Statistical significance *p* was estimated with the independent sample t-test for ADT and ANOVA for age groups

Baseline comparisons between the age groups in Fig. 2 revealed high urinary incontinence, urinary irritation and obstruction, bowel function and hormonal function across age groups. The statistically significant (*p* = 0.001) and potentially clinically relevant difference of 10–13 points (4–6 is considered clinically relevant [30]) was recorded in bowel function, where the mean scores for men < 65 and 65–74 years of age were 90 (95% CI 83 to 96) and 93 (95% CI 90 to 96) respectively, while for men in the ≥ 75 age group, it was 80 (95% CI 72 to 88). EPIC-26 scores were significantly lower for men on ADT than for men who did not receive ADT for urinary irritation and obstruction, bowel function and hormonal function domains (85 [95% CI 81 to 89] vs 92 [95% CI 90 to 95] (*p* = 0.003), 83 [95% CI 78 to 88] vs 95 [95% CI 92 to 97] (*p* < 0.001) and 67 [95% CI 62 to 73] vs 83 [95% CI 79 to 87] (*p* < 0.001), respectively). The respective 7, 12 and 16-point differences were also clinically significant [30]. In contrast, urinary incontinence was higher for men on ADT as compared to those not on ADT (*p* < 0.001), and the 17-point difference (90 [95% CI 86 to 94] vs 73 [95% CI 66 to 81]) was much higher than the previously estimated minimal clinically important difference for urinary incontinence of 6–9 points. Sexual function was low across the age groups, irrespective of ADT treatment.

**Fig. 3:**
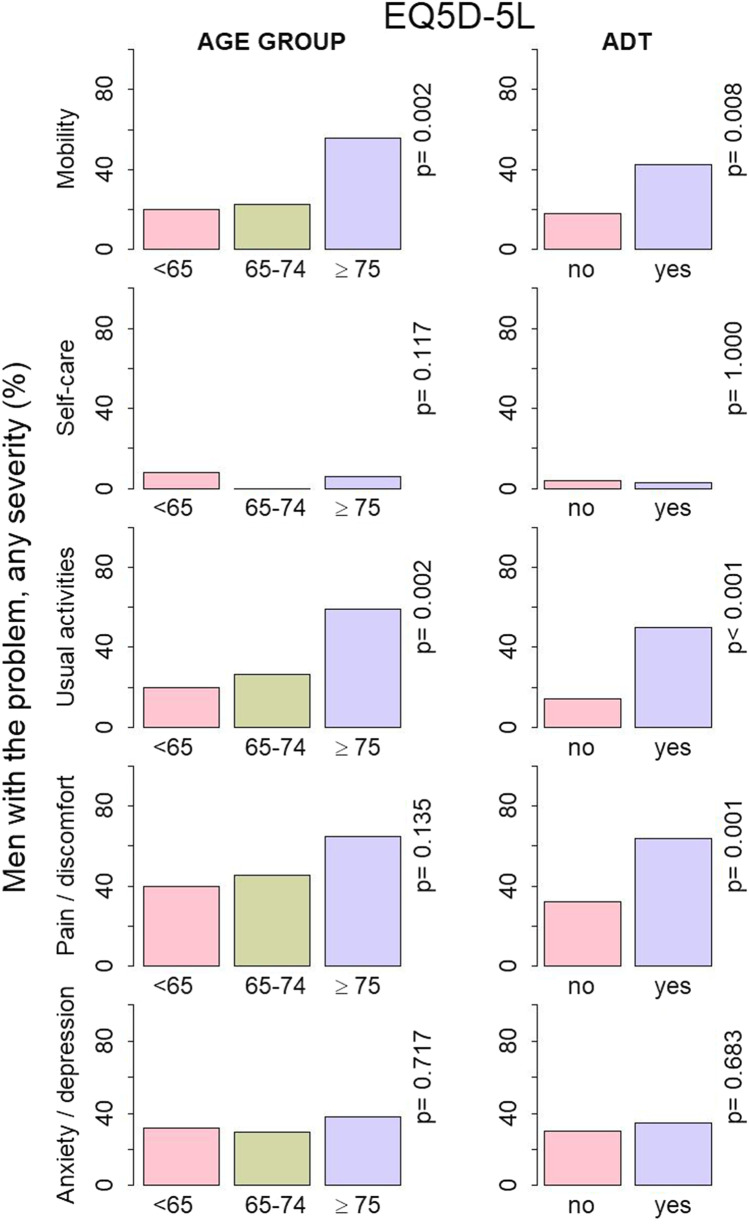
Baseline percentages of men experiencing the problem of any severity in the five EQ5D-5L domains: mobility, self-care, usual activities, pain and discomfort and anxiety and depression by age group and by androgen deprivation therapy (ADT) (yes/no). Statistical significance *p* was estimated with the Chi-squared test

The mean EQ5D-5L summary index value for the sample was 0.891 (95% CI 0.865 to 0.917) at baseline and 0.903 (95% CI 0.878 to 0.928) at 6 months (Table 2). The percentage of men reporting a full health state at baseline was 35% (95% CI 25 to 46) and 40% (95% CI 30 to 50) at 6 months. Over time, there were no statistically significant improvements in any of the EQ5D-5L domains. The percentage of men reporting problems of any severity with self-care, both at baseline and at 6 months, was low in comparison with other domains of EQ5D-5L (3%, 95% CI 1 to 9). This was also consistent across age groups and ADT treatments (Fig. 3). In contrast, pain and discomfort were reported by 50% of men (95% CI 39 to 60) at baseline and 48% (95% CI 38 to 59) at the 6-month follow-up (*p* = 1.000). There was also no difference in the prevalence of pain and discomfort between the age groups (*p* = 0.135), although more men who received ADT reported pain and discomfort than those not on ADT (*p* = 0.001). More mobility problems were reported by men in the ≥ 75 age group (*p* = 0.002) and by those on ADT (*p* = 0.008). The trend was similar in the usual activities domain, with more men in the ≥ 75 age group (*p* = 0.002) and on ADT (*p* < 0.001) reporting a problem than those in other groups.

## Discussion

This study provides new insights into the impact of a community pharmacy-based lifestyle intervention on important health-related quality of life domains in men treated for prostate cancer. Lifestyle interventions have been shown to improve patient activation [31], quality of life and prostate cancer-specific function [32]. However, despite these potential benefits, they are rarely implemented as part of pre- and post-treatment rehabilitation services. In the present study, the community pharmacy intervention led to a significant improvement of 4 points in the PAM score. Previous research has shown that even a 1-point improvement in the latter is clinically relevant and that each point increase in PAM score correlates to a 2% decrease in hospitalization and 2% increase in medication adherence [29].

PAM has been widely adopted, especially in the UK and USA, and it has been used as an outcome measure to evaluate the effectiveness of healthcare interventions [33]. A substantial body of evidence exists to support the use of the PAM in clinical practice for the assessment and targeting of patients that require more support [34]. A recently published systematic review found that the level of PAM was associated with a variety of clinical indicators (e.g. BMI), health outcomes and behaviours [33]. It was also shown that PAM is a significant predictor of healthcare service utilisation and that less activated patients have higher healthcare costs. However, more research is needed to investigate how interventions could increase the levels of patient activation and how this could be sustained over time [35]. Healthcare interventions should be tailored to the individual ability and the level of patient activation. This applies especially to exercise interventions, where there is variation in the age, body strength and fitness of participants [14]. In this context, PAM score could be used to assess patients and encourage realistic behaviour change; i.e. the advice to people on PAM level 1 will be different to those on level 4 [34].

In addition to the improvement in PAM, there were significant improvements in physical activity and adherence to a Mediterranean diet, both of which could have potential clinical relevance in reducing obesity and cardiovascular risk [36, 37]. However, there was no evidence that the intervention improved function or quality of life. Men reported good functional outcomes across all EPIC-26 domains except the sexual domain. Poor sexual function was common across all age groups, irrespective of ADT. The largest population-based study to date, published in 2019 [38], which included more than 30,000 UK men with prostate cancer, reported a similar high prevalence of sexual dysfunction and that half of the men were not offered any support for this important health issue. An Australian population-based study [39], published in 2020, also showed high levels of sexual dysfunction in this population, which persisted for 15 years after treatment for cancer. Sexual function and other quality of life outcomes were also shown to be worse in men with prostate cancer as compared to age-matched controls [39].

In the present study, subgroup analysis revealed some important age-related differences in the prevalence of impaired functional outcomes. For example, older men revealed more problems with mobility and usual activities than younger men. Although other studies that report PROs in men with prostate cancer report similar results, this is not a prostate cancer-specific finding, since older people generally report more problems in all the EQ5D-5L domains, with the strongest effect of age in the mobility domain [40]. However, the differences in mean scores for the EPIC-26 domains found in this study between the ADT subgroups did not seem to be age-related. This is similar to other studies [1, 38, 41], where men treated with ADT reported worse functional outcomes than those who did not receive ADT.

### Strengths and limitations

To our knowledge, this is the only study to date that reports patient activation, lifestyle habits such as diet and exercise and prostate cancer-specific quality of life and functional outcomes before and after men participated in a community pharmacy lifestyle intervention. We used a standard set of validated and internationally recognised questionnaires as defined by the working group of the International Consortium for Health Outcomes Measurement (ICHOM) [16]. This is a key strength of the study, as it allows international comparisons. The intervention led to significant improvements in patient activation, exercise, dietary habits and some functional outcomes. We also report the effects of age and ADT on outcomes. These data are needed to understand more about the burden for men affected by prostate cancer who live in the community, and the scale of the problem in this group of patients. It is important to note that ADT could be correlated with other variables such as age or treatment (i.e. radiotherapy vs surgery). Therefore, the differences observed between the ADT groups could be due to age or other treatment modalities.

The study was a non-randomised, one arm trial, in which all participants received the intervention, so there was no control group. In addition, the study sample was relatively small and heterogeneous, as it included men, irrespective of their treatment modality, and at varying times post-treatment. More definitive, randomised controlled trials with larger sample sizes are required to assess the effectiveness of this intervention. Finally, while we report changes in outcomes from baseline for up to 6 months, assessments beyond 6 months would be needed to determine long-term, lasting changes.

## Conclusions

The study showed that community pharmacy intervention can lead to significant improvements in patient activation, exercise and dietary habits. The encouraging results warrant a definitive randomised controlled trial (RCT) to assess the effectiveness of the intervention. The study also showed that sexual dysfunction, pain and discomfort are common after treatment for localised prostate cancer. Pre- and post-treatment rehabilitation approaches are clearly needed to support prostate cancer patients living with long-term health problems. With the expanding role of the community pharmacy in long-term management of chronic conditions in the UK and worldwide, this study shows how the community pharmacy could play a key role in the context of prostate cancer management and rehabilitation. With a high proportion of men living many years after prostate cancer, community pharmacy could be the first point of contact for some of the quality of life and functional problems.

## Supplementary Information

Below is the link to the electronic supplementary material.

Supplementary file1 (PDF 334 kb)

Supplementary file2 (PDF 301 kb)

Supplementary file3 (PDF 459 kb)

## Data Availability

The data can be requested by contacting corresponding author. The access will be granted subject to a reasonable request and data sharing agreement.

## Code availability

No bespoke software was created.
