# Supplementary material for: Patient activation and patient-reported outcomes of men from a community pharmacy lifestyle intervention after prostate cancer treatment
Source: Support Care Cancer. 2021 Jul 21;30(1):347–58. doi: 10.1007/s00520-021-06404-5 (PMC8636444; doi:10.1007/s00520-021-06404-5)
Supplement: Supplementary file 1 — Supplementary file1 (PDF 334 kb) [file 520_2021_6404_MOESM1_ESM.pdf]

True NTH Exercise and Diet Project
Baseline Questionnaire

Thank you for agreeing to take part in the True NTH Exercise & Diet Project: Phase 2 project.

Before you attend your first pharmacy appointment, we would like you to answer the questions included in this pack. Please read each question carefully. There are no right or wrong answers to these questions.

You may find some of the questions feel rather personal. However, it is important we ask about these issues so that we can understand some of the problems experienced by men. If you decide there are any questions you would rather not answer, then please leave it blank and go on to the next question.

The questions should take no more than 15 minutes to complete. When you have worn your Actiwatch for seven days, please could you put it, together with this completed questionnaire in the freepost envelope provided. *(It does not matter when you complete the questionnaire during the week).*

All the information you give will be treated as strictly confidential and will only be seen by the University of Surrey True NTH Exercise & Diet Project team. None of the information will be shared with your GP, hospital consultant or community pharmacy team.

If you have any questions about any aspect of completing the questionnaire, or if you require the questionnaire to be supplied in a different format (e.g. large print) please contact Dr Karen Poole (Research Fellow).

Karen can be reached on 01483 686741 or by email k.poole@surrey.ac.uk

Participant ID Number:

Please complete today's date:

PAM

Below are some statements that people sometimes make when they talk about their health.

Please indicate **how much you agree or disagree** with each statement as it applies to you personally by ticking your answer. Your answers should be what is true for you and not just what you think others want you to say.

If the statement does not apply to you, tick N/A	Disagree Strongly	Disagree	Agree	Agree Strongly	N/A
1. When all is said and done, I am the person who is responsible for taking care of my health					
2. Taking an active role in my own health care is the most important thing that affects my health					
3. I am confident I can help prevent or reduce problems associated with my health					
4. I know what each of my prescribed medications do					
5. I am confident that I can tell whether I need to go to the doctor or whether I can take care of a health problem myself					
6. I am confident that I can tell a doctor concerns I have even when he or she does not ask					
7. I am confident that I can follow through on medical treatments I may need to do at home					
8. I understand my health problems and what causes them					
9. I know what treatments are available for my health problems					
10. I have been able to maintain (keep up with) lifestyle changes, like eating right or exercising					
11. I know how to prevent problems with my health					
12. I am confident I can figure out solutions when new problems arise with my health					
13. I am confident that I can maintain lifestyle changes, like eating right and exercising, even during times of stress					

Under each heading, please tick the ONE box that best describes your health TODAY:

MOBILITY

- ☐ I have no problems in walking about
- ☐ I have slight problems in walking about
- ☐ I have moderate problems in walking about
- ☐ I have severe problems in walking about
- ☐ I am unable to walk about

SELF-CARE

- ☐ I have no problems washing or dressing myself
- ☐ I have slight problems washing or dressing myself
- ☐ I have moderate problems washing or dressing myself
- ☐ I have severe problems washing or dressing myself
- ☐ I am unable to wash or dress myself

USUAL ACTIVITIES
(e.g. work, study, housework, family or leisure activities)

- ☐ I have no problems doing my usual activities
- ☐ I have slight problems doing my usual activities
- ☐ I have moderate problems doing my usual activities
- ☐ I have severe problems doing my usual activities
- ☐ I am unable to do my usual activities

PAIN/DISCOMFORT

- ☐ I have no pain or discomfort
- ☐ I have slight pain or discomfort
- ☐ I have moderate pain or discomfort
- ☐ I have severe pain or discomfort
- ☐ I have extreme pain or discomfort

ANXIETY /DEPRESSION

- ☐ I am not anxious or depressed
- ☐ I am slightly anxious or depressed
- ☐ I am moderately anxious or depressed
- ☐ I am severely anxious or depressed
- ☐ I am extremely anxious or depressed

We would like to know how good or bad your health is TODAY.

This scale is numbered from 0 to 100.

100 means the <u>best</u> health you can imagine.
0 means the <u>worst</u> health you can imagine.

Mark an X on the scale to indicate how your health is TODAY.

Now, please write the number you marked on the scale in the box below.

Your health today is

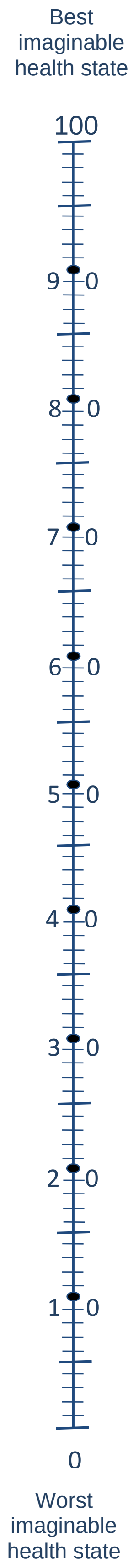

ABOUT YOUR SYMPTOMS

EPIC- 26

This questionnaire is designed to measure Quality of Life issues in patients with prostate cancer. To help us get the most accurate measurement, it is important that you answer all questions honestly and completely. Remember, as with all medical records, information contained within this survey will remain strictly confidential.

These questions are about your urinary habits. Please consider ONLY THE LAST FOUR WEEKS

1. Over the **past four weeks**, how often have you leaked urine?
Please tick one

- ☐ More than once a day
- ☐ About once a day
- ☐ More than once a week
- ☐ About once a week
- ☐ Rarely or never

2. Which of these best describes your urinary control during the **last four weeks?**
Please tick one

- ☐ No urinary control whatsoever
- ☐ Frequent dribbling
- ☐ Occasional dribbling
- ☐ Total urinary control

3. During the **last four weeks,** how many pads have you been using to control leakage?
Please tick one

- ☐ None
- ☐ One pad per day
- ☐ Two pads per day
- ☐ Three or more pads per day

4. How big a problem, if any, has each of the following been for you during the **last four weeks**? *Tick one box on each row*

	No problem	very small problem	small problem	moderate problem	big problem
a. Dripping or leaking urine					
b. Pain or burning on urination					
c. Bleeding with urination					
d. Weak urine stream/incomplete emptying					
e. Needing to urinate frequently in the day					

5. Overall, how big a problem has your urinary function been for you during the **last four weeks**?
Please tick one

- ☐ No problem
- ☐ Very small problem
- ☐ Small problem
- ☐ Moderate problem
- ☐ Big problem

6. How big a problem, if any, has each of the following been for you during the **last four** weeks? *Tick one box on each row*	No problem	very small problem	small problem	moderate problem	big problem
a. Urgency to have a bowel movement					
b. Increased frequency of bowel movements					
c. Watery bowel movements					
d. Losing control of your bowel movements					
e. Blood in your stools/bowel movements					
f. Abdominal, pelvic or rectal pain					

7. Overall, how big a problem have your bowel habits been for you during the **last four weeks?**

Please tick one

- ☐ No problem
- ☐ Very small problem
- ☐ Small problem
- ☐ Moderate problem
- ☐ Big problem

The next few questions ask about your current sexual function and sexual satisfaction.

Many of the questions are very personal, but your answers will help us understand the important issues that you face every day. Remember that this survey information is **COMPLETELY CONFIDENTIAL**. Please answer honestly about the last **FOUR WEEKS ONLY**

8. How would you rate each or the following during **the last four weeks**? *Tick one on each row*	Very poor to none	Poor	Fair	Good	Very good
a. Your ability to have an erection					
b. Your ability to reach orgasm (climax)					

9. How would you describe the usual **quality** of your erections during the **last four weeks**?

Please tick one

- ☐ No erection at all
- ☐ Not firm enough for any sexual activity
- ☐ Firm enough for foreplay or masturbation
- ☐ Firm enough for intercourse

10. How would you describe the **frequency** of your erections during the **last four weeks**?
Please tick one

- ☐ I **never** had an erection when I wanted one
- ☐ I had an erection **less than half** the time I wanted one
- ☐ I had an erection **about half** the time I wanted one
- ☐ I had an erection **more than half** the time I wanted one
- ☐ I had an erection **whenever** I wanted one

11. Overall, how would you rate your ability to function sexually over the **last four weeks**?
Please tick one

- ☐ Very poor
- ☐ Poor
- ☐ Fair
- ☐ Good
- ☐ Very good

12. Overall, how big a problem has your sexual function sexually or lack of sexual function been for you?
Please tick one

- ☐ No problem
- ☐ Very small problem
- ☐ Small problem
- ☐ Moderate problem
- ☐ Big problem

The next section is about your hormonal function. Please consider the last **FOUR WEEKS ONLY.**

13. How big a problem, if any, has each of the following been for you during the last **four weeks**? *Tick one on each row*

	No problem	very small problem	small problem	moderate problem	big problem
a. Hot flushes					
b. Breast tenderness/enlargement					
c. Loss of body hair					
d. Feeling depressed					
e. Lack of energy					
f. Change in body weight					

Thank you for taking the time to complete this questionnaire.

When you have worn your Actiwatch for seven days, please could you put it, together with this completed questionnaire in the freepost envelope provided.
