# Supplementary material for: Patient activation and patient-reported outcomes of men from a community pharmacy lifestyle intervention after prostate cancer treatment
Source: Support Care Cancer. 2021 Jul 21;30(1):347–58. doi: 10.1007/s00520-021-06404-5 (PMC8636444; doi:10.1007/s00520-021-06404-5)
Supplement: Supplementary file 2 — Supplementary file2 (PDF 301 kb) [file 520_2021_6404_MOESM2_ESM.pdf]

True NTH Exercise and Diet Project
3 Month Evaluation Questionnaire

Thank you for agreeing to take part in the True NTH Exercise & Diet Project: Phase 2 project.

Now that you have completed your health and fitness assessments,
we would like you to answer the questions enclosed.

Please read each question carefully. There are no right or wrong answers to these questions.
The questions should take no more than 20 minutes to complete.

When you have worn your Actiwatch for seven days, please could you put it,
together with this completed questionnaire and log in the freepost envelope provided.
(It does not matter when you complete the questionnaire during the week).

All the information you give will be treated as strictly confidential and will only be seen by the University of Surrey True NTH Exercise & Diet Project team.
None of the information will be shared with your GP, hospital consultant or community pharmacy team.

If you have any questions about any aspect of completing the questionnaire, or if you require the questionnaire to be supplied in a different format (e.g. large print) please contact Dr Karen Poole (Research Fellow).

Karen can be reached on 01483 686741 or by email k.poole@surrey.ac.uk

Participant ID Number:

Please complete today's date:

Below are some statements that people sometimes make when they talk about their health.

Please indicate **how much you agree or disagree** with each statement as it applies to you personally by ticking your answer. Your answers should be what is true for you and not just what you think others want you to say.

If the statement does not apply to you, tick N/A	Disagree Strongly	Disagree	Agree	Agree Strongly	N/A
1. When all is said and done, I am the person who is responsible for taking care of my health					
2. Taking an active role in my own health care is the most important thing that affects my health					
3. I am confident I can help prevent or reduce problems associated with my health					
4. I know what each of my prescribed medications do					
5. I am confident that I can tell whether I need to go to the doctor or whether I can take care of a health problem myself					
6. I am confident that I can tell a doctor concerns I have even when he or she does not ask					
7. I am confident that I can follow through on medical treatments I may need to do at home					
8. I understand my health problems and what causes them					
9. I know what treatments are available for my health problems					
10. I have been able to maintain (keep up with) lifestyle changes, like eating right or exercising					
11. I know how to prevent problems with my health					
12. I am confident I can figure out solutions when new problems arise with my health					
13. I am confident that I can maintain lifestyle changes, like eating right and exercising, even during times of stress					

The questions in this section are designed to help us understand the quality of your diet.
These questions will indicate how much each of the following foods/food groups/nutrients feature within your diet: saturated fat, unsaturated fat, vegetables and fruit, sugar, alcohol, pulses, fish, confectionery and protein.

You may remember that the University of Surrey Research team asked you these questions over the phone before your first pharmacy appointment and this time we would like you to complete the questions at home.

1.	Each week, which do you do more?<br>*Please tick one*	☐<br>☐	Eat from take-away or sit-in restaurants<br>Buy and cook your own food
2.	Do you usually feel well enough to shop for your food and cook it?<br>*Please tick one*	☐<br>☐	Yes<br>No
3.	Each week, how often do you skip breakfast?<br>*Please tick one*	☐<br>☐<br>☐	Usually/often<br>Sometimes<br>Rarely/never
4.	How often do you eat fried foods such as fried meat or fish, fried eggs, fried chips or deep-fried cheese each week?<br>*Please tick one*	☐<br>☐<br>☐	Usually/often<br>Sometimes<br>Rarely/never
5.	How often do you eat processed meats per week?<br>*(This includes burgers, bacon, sausages, hot-dogs, corned-beef, salami or other cured meats but NOT low-fat or reduced-fat varieties or normal sliced meat)*<br>*Please tick one*	☐<br>☐<br>☐	Usually/often<br>Sometimes<br>Rarely/never
6.	Do you use olive oil as your main source of fat for cooking?<br>*Please tick one*	☐<br>☐	Yes<br>No
7.	How much olive oil do you consume per day?<br>*(This includes olive oil that you use in cooking, on salads and in take-away or restaurant meals)*<br>*Please tick one*	☐<br>☐	Less than 4 tablespoons<br>4 tablespoons or more
8.	How many servings of vegetables do you consume per day?<br>*(Please count a serving as 80g/3oz which equates to 2-3 tablespoons of vegetables)*<br>*Please tick one*	☐<br>☐	Less than 5 portions<br>5 or more portions

9.	How many pieces of fruit do you consume per day?<br>*(one piece is up to one glass of unsweetened fruit juice; one medium sized piece of fruit e.g. apple; 2-3 smaller pieces of fruit e.g. plums; a handful of grapes or berries)*<br>*Please tick one*	☐<br>☐	Less than 3 pieces<br>3 pieces or more
10.	How many servings of red meat, burgers or sausages do you consume per day?<br>*(A serving is 100-150g/3.5-5.3oz) (1-1.5 decks of cards, quarter pound burger, 1-2 sausages)*<br>*Please tick one*	☐<br>☐	Less than 1<br>1 or more
11.	How many servings of butter, margarine (12g/0.4oz) or cream do you consume per day?<br>*(12g = thick spread of butter on a slice of bread)*<br>*Please tick one*	☐<br>☐	Less than 1<br>1 or more
12.	How many sweetened drinks such as fruit squash and fizzy drinks do you consume per day?<br>*(Please do not count no-added sugar squash or diet drinks)*<br>*Please tick one*	☐<br>☐	Less than 1<br>1 or more
13.	If you drink red or white wine, how much do you consume per week?<br>(Please note a bottle is 750ml)<br>*Please tick one*	☐<br>☐	Less than 750ml per week<br>More than or equal to 750ml per week
14.	How many servings of pulses such as lentils, beans etc. do you consume per week?<br>*(A serving is 3-4 tablespoons)*<br>*Please tick one*	☐<br>☐	Less than 3<br>3 or more
15.	How many servings of fish/seafood do you consume per week?<br>*(A serving is roughly 100-150g/3.5-5.3oz fish or 200g/7oz shellfish such as prawns)*<br>*Please tick one*	☐<br>☐	Less than 3<br>3 or more
16.	How often do you consume biscuits, cakes or pastries each week?<br>*Please tick one*	☐<br>☐	2 times or less<br>More than 2 times

17.	How many servings of nuts do you eat per week?<br>*(A serving is roughly 30g or a small handful)*<br>*Please tick one*	☐<br>☐	Less than 3 times<br>3 times or more
18.	Do you prefer to eat white meats such as chicken, turkey and rabbit or dark meats such as beef, pork, hamburgers or sausages?<br>*Please tick one*	☐<br>☐	White meats<br>Dark meats
19.	How many times per week do you eat dishes which contain a tomato based sauce?<br>*Please tick one*	☐<br>☐	Less than 2 times<br>2 times or more

Thank you for taking the time to complete these questions,
your contribution to this research project is extremely valuable.

When you have worn your Actiwatch for seven days,
please could you put the following into the freepost envelope and return it to us at the University of Surrey;

- This completed questionnaire
- Your completed Actiwatch log
- Your worn Actiwatch

Thank you from Sara, Karen, Sophie and Fiona at the University of Surrey TrueNTH Research team!
