# Supplementary material for: Patient activation and patient-reported outcomes of men from a community pharmacy lifestyle intervention after prostate cancer treatment
Source: Support Care Cancer. 2021 Jul 21;30(1):347–58. doi: 10.1007/s00520-021-06404-5 (PMC8636444; doi:10.1007/s00520-021-06404-5)
Supplement: Supplementary file 3 — Supplementary file3 (PDF 459 kb) [file 520_2021_6404_MOESM3_ESM.pdf]

True NTH Exercise and Diet Project
6 Month Evaluation Questionnaire

Thank you for agreeing to take part in the True NTH Exercise & Diet Project: Phase 2 project.

It is now six months since you started this project so we would like you to answer the questions included in this pack. Some of these questions you will have completed before, and we are interested in your responses today. Please read each question carefully. There are no right or wrong answers to these questions.

You may find some of the questions feel rather personal. However, it is important we ask about these issues so that we can understand some of the problems experienced by men. If you decide there are any questions you would rather not answer, then please leave it blank and go on to the next question.

The questions should take no more than 35 minutes to complete. When you have worn your Actiwatch for seven days, please could you put it, together with this completed questionnaire in the freepost envelope provided. *(It does not matter when you complete the questionnaire during the week).*

All the information you give will be treated as strictly confidential and will only be seen by the University of Surrey True NTH Exercise & Diet Project team. None of the information will be shared with your GP, hospital consultant or community pharmacy team.

If you have any questions about any aspect of completing the questionnaire, or if you require the questionnaire to be supplied in a different format (e.g. large print) please contact Dr Karen Poole (Research Fellow).

Karen can be reached on 01483 686741 or by email k.poole@surrey.ac.uk

Participant ID Number:

Please complete today's date:

Below are some statements that people sometimes make when they talk about their health.

Please indicate **how much you agree or disagree** with each statement as it applies to you personally by ticking your answer. Your answers should be what is true for you and not just what you think others want you to say.

If the statement does not apply to you, tick N/A

		Disagree Strongly	Disagree	Agree	Agree Strongly	N/A
1.	When all is said and done, I am the person who is responsible for taking care of my health					
2.	Taking an active role in my own health care is the most important thing that affects my health					
3.	I am confident I can help prevent or reduce problems associated with my health					
4.	I know what each of my prescribed medications do					
5.	I am confident that I can tell whether I need to go to the doctor or whether I can take care of a health problem myself					
6.	I am confident that I can tell a doctor concerns I have even when he or she does not ask					
7.	I am confident that I can follow through on medical treatments I may need to do at home					
8.	I understand my health problems and what causes them					
9.	I know what treatments are available for my health problems					
10.	I have been able to maintain (keep up with) lifestyle changes, like eating right or exercising					
11.	I know how to prevent problems with my health					
12.	I am confident I can figure out solutions when new problems arise with my health					
13.	I am confident that I can maintain lifestyle changes, like eating right and exercising, even during times of stress					

Under each heading, please tick the ONE box that best describes your health TODAY:

**MOBILITY**	☐	I have no problems in walking about
	☐	I have slight problems in walking about
	☐	I have moderate problems in walking about
	☐	I have severe problems in walking about
	☐	I am unable to walk about
**SELF-CARE**	☐	I have no problems washing or dressing myself
	☐	I have slight problems washing or dressing myself
	☐	I have moderate problems washing or dressing myself
	☐	I have severe problems washing or dressing myself
	☐	I am unable to wash or dress myself
**USUAL ACTIVITIES** *(e.g. work, study, housework, family or leisure activities)*	☐	I have no problems doing my usual activities
	☐	I have slight problems doing my usual activities
	☐	I have moderate problems doing my usual activities
	☐	I have severe problems doing my usual activities
	☐	I am unable to do my usual activities
**PAIN/DISCOMFORT**	☐	I have no pain or discomfort
	☐	I have slight pain or discomfort
	☐	I have moderate pain or discomfort
	☐	I have severe pain or discomfort
	☐	I have extreme pain or discomfort
**ANXIETY /DEPRESSION**	☐	I am not anxious or depressed
	☐	I am slightly anxious or depressed
	☐	I am moderately anxious or depressed
	☐	I am severely anxious or depressed
	☐	I am extremely anxious or depressed

Q5D-5L

We would like to know how good or bad your health is TODAY.

This scale is numbered from 0 to 100.

100 means the <u>best</u> health you can imagine.
0 means the <u>worst</u> health you can imagine.

Mark an X on the scale to indicate how your health is TODAY.

Now, please write the number you marked on the scale in the box below.

Your health today is

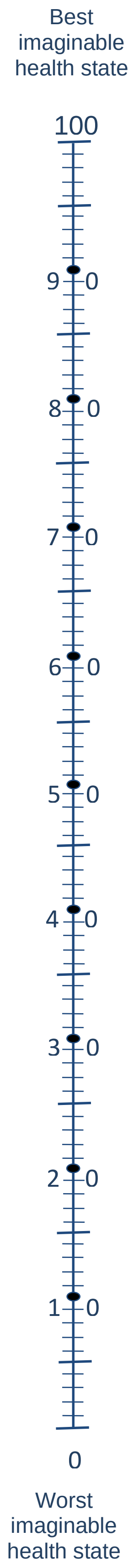

ABOUT YOUR SYMPTOMS

EPIC- 26

This questionnaire is designed to measure Quality of Life issues in patients with prostate cancer. To help us get the most accurate measurement, it is important that you answer all questions honestly and completely. Remember, as with all medical records, information contained within this survey will remain strictly confidential.

These questions are about your urinary habits. Please consider ONLY THE LAST FOUR WEEKS

1. Over the **past four weeks**, how often have you leaked urine?
Please tick one

- ☐ More than once a day
- ☐ About once a day
- ☐ More than once a week
- ☐ About once a week
- ☐ Rarely or never

2. Which of these best describes your urinary control during the **last four weeks?**
Please tick one

- ☐ No urinary control whatsoever
- ☐ Frequent dribbling
- ☐ Occasional dribbling
- ☐ Total urinary control

3. During the **last four weeks,** how many pads have you been using to control leakage?
Please tick one

- ☐ None
- ☐ One pad per day
- ☐ Two pads per day
- ☐ Three or more pads per day

4. How big a problem, if any, has each of the following been for you during the **last four weeks**? *Tick one box on each row*

	No problem	very small problem	small problem	moderate problem	big problem
a. Dripping or leaking urine					
b. Pain or burning on urination					
c. Bleeding with urination					
d. Weak urine stream/incomplete emptying					
e. Needing to urinate frequently in the day					

5. Overall, how big a problem has your urinary function been for you during the **last four weeks**?
Please tick one

- ☐ No problem
- ☐ Very small problem
- ☐ Small problem
- ☐ Moderate problem
- ☐ Big problem

6. How big a problem, if any, has each of the following been for you during the **last four** weeks? *Tick one box on each row*

	No problem	very small problem	small problem	moderate problem	big problem
a. Urgency to have a bowel movement					
b. Increased frequency of bowel movements					
c. Watery bowel movements					
d. Losing control of your bowel movements					
e. Blood in your stools/bowel movements					
f. Abdominal, pelvic or rectal pain					

7. Overall, how big a problem have your bowel habits been for you during the **last four weeks?**
Please tick one

- ☐ No problem
- ☐ Very small problem
- ☐ Small problem
- ☐ Moderate problem
- ☐ Big problem

The next few questions ask about your current sexual function and sexual satisfaction.

Many of the questions are very personal, but your answers will help us understand the important issues that you face every day. Remember that this survey information is **COMPLETELY CONFIDENTIAL**. Please answer honestly about the last **FOUR WEEKS ONLY**

8. How would you rate each or the following during **the last four weeks**?
Tick one on each row

	Very poor to none	Poor	Fair	Good	Very good
a. Your ability to have an erection					
b. Your ability to reach orgasm (climax)					

9. How would you describe the usual **quality** of your erections during the **last four weeks**?
Please tick one

- ☐ No erection at all
- ☐ Not firm enough for any sexual activity
- ☐ Firm enough for foreplay or masturbation
- ☐ Firm enough for intercourse

10. How would you describe the **frequency** of your erections during the **last four weeks**?
Please tick one

- ☐ I **never** had an erection when I wanted one
- ☐ I had an erection **less than half** the time I wanted one
- ☐ I had an erection **about half** the time I wanted one
- ☐ I had an erection **more than half** the time I wanted one
- ☐ I had an erection **whenever** I wanted one

11. Overall, how would you rate your ability to function sexually over the **last four weeks**?
Please tick one

- ☐ Very poor
- ☐ Poor
- ☐ Fair
- ☐ Good
- ☐ Very good

12. Overall, how big a problem has your sexual function sexually or lack of sexual function been for you?
Please tick one

- ☐ No problem
- ☐ Very small problem
- ☐ Small problem
- ☐ Moderate problem
- ☐ Big problem

The next section is about your hormonal function. Please consider the last **FOUR WEEKS ONLY.**

13. How big a problem, if any, has each of the following been for you during the last **four weeks**? *Tick one on each row*

	No problem	very small problem	small problem	moderate problem	big problem
a. Hot flushes					
b. Breast tenderness/enlargement					
c. Loss of body hair					
d. Feeling depressed					
e. Lack of energy					
f. Change in body weight					

The questions in this section are designed to help us understand the quality of your diet.
These questions will indicate how much each of the following foods/food groups/nutrients feature within your diet: saturated fat, unsaturated fat, vegetables and fruit, sugar, alcohol, pulses, fish, confectionery and protein.

1.	Each week, which do you do more?<br>*Please tick one*	☐<br>☐	Eat from take-away or sit-in restaurants<br>Buy and cook your own food
2.	Do you usually feel well enough to shop for your food and cook it?<br>*Please tick one*	☐<br>☐	Yes<br>No
3.	Each week, how often do you skip breakfast?<br>*Please tick one*	☐<br>☐<br>☐	Usually/often<br>Sometimes<br>Rarely/never
4.	How often do you eat fried foods such as fried meat or fish, fried eggs, fried chips or deep-fried cheese each week?<br>*Please tick one*	☐<br>☐<br>☐	Usually/often<br>Sometimes<br>Rarely/never
5.	How often do you eat processed meats per week?<br>*(This includes burgers, bacon, sausages, hot-dogs, corned-beef, salami or other cured meats but NOT low-fat or reduced-fat varieties or normal sliced meat)*<br>*Please tick one*	☐<br>☐<br>☐	Usually/often<br>Sometimes<br>Rarely/never
6.	Do you use olive oil as your main source of fat for cooking?<br>*Please tick one*	☐<br>☐	Yes<br>No
7.	How much olive oil do you consume per day?<br>*(This includes olive oil that you use in cooking, on salads and in take-away or restaurant meals)*<br>*Please tick one*	☐<br>☐	Less than 4 tablespoons<br>4 tablespoons or more
8.	How many servings of vegetables do you consume per day?<br>*(Please count a serving as 80g/3oz which equates to 2-3 tablespoons of vegetables)*<br>*Please tick one*	☐<br>☐	Less than 5 portions<br>5 or more portions

9.	How many pieces of fruit do you consume per day?<br>*(one piece is up to one glass of unsweetened fruit juice; one medium sized piece of fruit e.g. apple; 2-3 smaller pieces of fruit e.g. plums; a handful of grapes or berries)*<br>*Please tick one*	☐<br>☐	Less than 3 pieces<br>3 pieces or more
10.	How many servings of red meat, burgers or sausages do you consume per day?<br>*(A serving is 100-150g/3.5-5.3oz) (1-1.5 decks of cards, quarter pound burger, 1-2 sausages)*<br>*Please tick one*	☐<br>☐	Less than 1<br>1 or more
11.	How many servings of butter, margarine (12g/0.4oz) or cream do you consume per day?<br>*(12g = thick spread of butter on a slice of bread)*<br>*Please tick one*	☐<br>☐	Less than 1<br>1 or more
12.	How many sweetened drinks such as fruit squash and fizzy drinks do you consume per day?<br>*(Please do not count no-added sugar squash or diet drinks)*<br>*Please tick one*	☐<br>☐	Less than 1<br>1. or more
13.	If you drink red or white wine, how much do you consume per week?<br>(Please note a bottle is 750ml)<br>*Please tick one*	☐<br>☐	Less than 750ml per week<br>More than or equal to 750ml per week
14.	How many servings of pulses such as lentils, beans etc. do you consume per week?<br>*(A serving is 3-4 tablespoons)*<br>*Please tick one*	☐<br>☐	Less than 3<br>3 or more
15.	How many servings of fish/seafood do you consume per week?<br>*(A serving is roughly 100-150g/3.5-5.3oz fish or 200g/7oz shellfish such as prawns)*<br>*Please tick one*	☐<br>☐	Less than 3<br>3 or more
16.	How often do you consume biscuits, cakes or pastries each week?<br>*Please tick one*	☐<br>☐	2 times or less<br>More than 2 times

17.	How many servings of nuts do you eat per week?<br>*(A serving is roughly 30g or a small handful)*<br>*Please tick one*	☐<br>☐	Less than 3 times<br>3 times or more
18.	Do you prefer to eat white meats such as chicken, turkey and rabbit or dark meats such as beef, pork, hamburgers or sausages?<br>*Please tick one*	☐<br>☐	White meats<br>Dark meats
19.	How many times per week do you eat dishes which contain a tomato based sauce?<br>*Please tick one*	☐<br>☐	Less than 2 times<br>2 times or more

We would like to know about ***the health services and other support you have received over the last 3 months*** *i.e. since you completed in the pharmacy the True NTH Exercise and Diet programme.*

We are interested in these areas:

1. *The Health care you have received from your GP or community services*
2. *The Hospital care you have received*
3. *The leisure and wider services you have used*
4. *The support you have received from others*
5. *Whether you would be willing to pay for such services*

1. Thinking about the health care you have received from your GP or community services.

In the last 3 months have you….

		**If YES**, how many times?	**If YES**, please tell us the reasons for the consultation(s): were they related to your prostate cancer, not about your prostate, or both?		
Visited your GP?	☐ No<br>☐Yes	…………times	☐ Prostate-related	☐ Not for prostate	☐Both
Had advice from a GP on the phone?	☐ No<br>☐ Yes	…………times	☐ Prostate-related	☐ Not for prostate	☐ Both
Been visited by a GP or out-of-hours doctor at home?	☐ No<br>☐ Yes	…………times	☐ Prostate-related	☐ Not for prostate	☐ Both
Seen a nurse at the GP surgery?	☐ No<br>☐ Yes	…………times	☐ Prostate-related	☐ Not for prostate	☐ Both
Had advice from a nurse over the phone?	☐ No<br>☐ Yes	…………times	☐ Prostate-related	☐ Not for prostate	☐ Both
Been visited at home by a nurse?	☐ No<br>☐ Yes	…………times	☐ Prostate-related	☐ Not for prostate	☐ Both
Visited any other health care professionals (e.g. physiotherapist, dietician)?	☐ No<br>☐ Yes	…………times	☐ Prostate-related	☐ Not for prostate	☐ Both
Had advice from other health care professionals by phone?	☐ No<br>☐Yes	…………times	☐ Prostate-related	☐ Not for prostate	☐ Both

**2. Thinking specifically about hospital care. In the last 3 months have you**					
		**If YES**, how many times?	**If YES**, please tell us the reasons for the consultation(s): where they related to your prostate cancer, not about your prostate, or both?		
Attended hospital clinic about your prostate cancer?	☐ No<br>☐ Yes	…………times	N/A	N/A	N/A
Seen a urology cancer nurse specialist about your prostate cancer?	☐ No<br>☐ Yes	…………times	N/A	N/A	N/A
Spoken to a urology cancer nurse specialist by phone about your prostate cancer?	☐ No<br>☐ Yes	…………times	N/A	N/A	N/A
Attended any outpatient clinics at the hospital for other physical health problems?	☐ No<br>☐ Yes	…………times	☐ Prostate-related	☐ Not for prostate	☐ Both
Attended any other clinics for;<br>Heart disease	☐ No<br>☐ Yes	…………times	☐ Prostate-related	☐ Not for prostate	☐ Both
Diabetes	☐ No<br>☐ Yes	…………times	☐ Prostate-related	☐ Not for prostate	☐ Both
Blood pressure treatment	☐ No<br>☐ Yes	…………times	☐ Prostate-related	☐ Not for prostate	☐ Both
Attended A&E?	☐ No<br>☐ Yes	…………times	☐ Prostate-related	☐ Not for prostate	☐ Both
Been in hospital as a day case *(i.e. assigned a bed but no overnight stay)*	☐ No<br>☐ Yes	…………times	☐ Prostate-related	☐ Not for prostate	☐ Both

**3. Thinking about how you may have used leisure and wider services.**					
**In the last 3 months have you…….**		**If YES,** How many times?	**If YES**, please tell us the reasons for the going: were they related to your pharmacy visits, or have always been going, or are you now doing more?		
Attended a gym or leisure centre?	☐ No ☐ Yes	…………times	☐ Pharmacy related	☐ Always been going	☐Doing more
Been swimming?	☐ No ☐ Yes	…………times	☐ Pharmacy related	☐ Always been going	☐Doing more
Been to a walking for health group?	☐ No ☐ Yes	…………times	☐ Pharmacy related	☐ Always been going	☐Doing more
Joined another fitness club?	☐ No ☐ Yes	…………times	☐ Pharmacy related	☐ Always been going	☐Doing more
Had "exercise on prescription" from your GP?	☐ No ☐ Yes	…………times	☐ Pharmacy related	☐ Always been going	☐Doing more
Joined a weight loss club?	☐ No ☐ Yes	…………times	☐ Pharmacy related	☐ Always been going	☐Doing more
Been walking more?	☐ No ☐ Yes	………..times	☐ Pharmacy related	☐ been going	☐Doing more

**4. Thinking about the support you have had from others. In the last 3 months, have you**…..		
Visited a helpline for support?	☐No ☐ Yes	**If YES**, which one?
Been to a prostate cancer support group?	☐No ☐Yes	**If YES**, which one?
Made contact with friends, acquaintances or colleagues to talk about exercise and diet and receive support?	☐No ☐ Yes	

5. It may be possible in the future to introduce True NTH Exercise and Diet pharmacy-led services more widely but currently this is funded by charities. We would like to ask about your willingness to pay if this was provided more widely.

5a. Would you choose to have the service if not provided for free?

☐ No (go onto Q5c)

☐ Yes (got to next question)

5b. *If you are willing to pay,* how much would you be prepared to pay for the True NTH Exercise and Diet pharmacy service? (*Remember this is an imaginary situation as you will* <u>*not*</u> *be asked to pay for the tests you have experienced).*

Tick **yes** for the amounts you would be willing to pay.

Tick **no** for the amounts you would <u>not</u> willing to pay.

Amount Willing to Pay		
£20	☐ Yes	☐ No
£40	☐ Yes	☐ No
£80	☐ Yes	☐ No
£100	☐ Yes	☐ No
£150	☐ Yes	☐ No
£200	☐ Yes	☐ No

Now CIRCLE the <u>maximum</u> amount you would be willing to pay.

5c. *If you are NOT willing to pay* for the True NTH Exercise and Diet pharmacy-led service what is your main reason for this *(please tick ONE box only)*?

It was not of value to me	☐
I cannot afford to pay	☐
I object to paying	☐
Or another reason (please state)	

5d. If you were able to have only one element out of the True NTH exercise and diet pharmacy-led service, what would it be?

AND how would you order the elements in terms of how helpful they have been to you?

	Please tick ONE box only	***Please rank what helped you the most.*** *Give the score of 1 to the most helpful element, the score of 2 to the second most helpful element and so on until you get to 6 for the least helpful element.*
First health and fitness assessment at pharmacy and personalised lifestyle prescription	☐	
Full Resource Pack (DVD, Manual, Pedometer, stretch bands)	☐	
DVD only	☐	
Manual only	☐	
Telephone calls from pharmacist	☐	
Second health and fitness assessment at pharmacy	☐	

6. Please answer the following questions about yourself. Although some of these questions seem rather personal the information given is confidential, will remain anonymous and will help us in our analysis.

6a. How old were you when you left full time education?

6b. Please estimate the annual income of your household *(Please tick one)*

- Less than £6,000 ☐
- £7000- £10,000 ☐
- £11,000-£15,000 ☐
- £16,000-£20,000 ☐
- £21,000-£25,000 ☐
- More than £25,000 ☐

6c. Whilst in the pharmacy for your visits did you buy anything? ☐ No ☐ Yes

If yes, what did you buy? ....

6d. Did you go to the pharmacy outside of your consultations? ☐ No ☐ Yes

If yes, why did you go to the pharmacy? ....

If yes, how many visits did you make? ....

Please use this space (and overleaf) to add any other comments you would like to make regarding your experience of True NTH Exercise and Diet Pharmacy-led Service?

Thank you for taking the time to complete these questions, your contribution to this research project is extremely valuable.

When you have worn your Actiwatch for seven days,
please could you put the following into the free post envelope and return it to us at the University of Surrey:

- this completed questionnaire
- your completed Actiwatch log
- your worn Actiwatch

Thank you from Sara, Sophie and Fiona at the University of Surrey TrueNTH Research Team!
